# The Multistage 20-Meter Shuttle Run Test Reference Values for Tibetan Children and Adolescents in Tibet, China

**DOI:** 10.3390/ijerph191912703

**Published:** 2022-10-04

**Authors:** Xiaojian Yin, Feng Zhang, Pengwei Sun, Yuan Liu, Yaru Guo

**Affiliations:** 1 Key Laboratory of Adolescent Health Assessment and Exercise Intervention of Ministry of Education, East China Normal University, Shanghai 200241, China; 2 College of Physical Education and Health, East China Normal University, Shanghai 200241, China

**Keywords:** cardiorespiratory fitness, 20 m SRT, high-altitude, Tibet, LMS

## Abstract

Objective: Cardiorespiratory fitness (CRF) reference data for Tibetan (Zang ethnicity) children and adolescents at high altitudes in Tibet of China are lacking. The present study aimed to develop sex- and age-specific 20mSRT norms for Chinese Tibetan children and adolescents at high altitudes. Method: A total of 4667 participants from Lhasa (3650 m), Nagqu (4500 m), and Amdo (4700 m) were selected by a stratified random cluster sampling method in two stages. The 20 m SRT test was used to estimate cardiorespiratory fitness. The 20 m SRT norms were developed by the lambda, mu, and sigma method (LMS). Results: The 20 m SRT laps, completed stages/minutes, and the speed at the last complete stage of Chinese Tibetan children and adolescents aged 7–18 years increased with age. Conclusion: Given the importance of CRF for children and adolescents’ health, the government should strengthen the monitoring of the CRF of Tibetan children and adolescents in high-altitude areas, strengthen physical education curriculum reform, and increase the level of physical activity in order to improve the level of CRF in children and adolescents.

## 1. Introduction

As an important indicator of health and a core element of physical fitness, cardiorespiratory fitness (CRF) comprehensively reflects the ability to absorb, transport, and use oxygen [1]. Substantial epidemiological and clinical evidence has suggested that lower CRF is strongly associated with an increased risk of cardiovascular disease [1,2], all-cause mortality [3], and cancer mortality [4]. Moreover, low CRF is regarded as an independent all-cause mortality indicator [5] and considered to be more reliable than traditional predictors, such as hypertension, smoking, type 2 diabetes, and hyperlipidemia [6]. Unfortunately, only two-thirds of the boys (mean ± 95%CI: 67 ± 14%) and half of the girls (mean ± 95% CI: 54 ± 17%) in the world are believed to have healthy CRF [7]. In China, the CRF of children and adolescents significantly declined from 1995 to 2014 (*p* < 0.0001) [8].

The 20-m shuttle run test (20-m SRT), a simple and cost-effective test, can test large groups of children simultaneously [9,10]. It was reported that the 20-m SRT was highly correlated with VO_2max_ in children and adolescents (R = 0.91, *p* < 0.05) [11]. Additionally, Leger et al. reported a test–retest reliability coefficient of 0.89 for the 20-m SRT in children [12]. Hence, it is recognized as an effective field-based measurement of CRF for children and adolescents [13] and has been widely used worldwide [10,14,15].

Assessing CRF from an early age may help to identify the target population for primary prevention as well as be helpful for health promotion policies. The key to scientifically and accurately assessing CRF lies in developing normative values. International sex- and age-specific 20 m SRT norms based on 1,142,026 children and adolescents from 50 countries were established for health and fitness screening and monitoring [7]. Furthermore, 20-m SRT norms for regional children and adolescents from areas such as Europe [16], North America [17], Oceania [18], and England [19,20] have been reported as well. We also previously reported normative reference values for Chinese children and adolescents [21]. However, 20-m SRT reference data for Tibetan (Zang ethnicity) children and adolescents at high altitudes in Tibet, China, are lacking.

Known as the “roof of the world”, the Tibet Plateau has a typical plateau climate and is home to about 5 million Tibetans, with 66% of them living at altitudes above 3500 m, and with ancestors having lived at similar altitudes for more than 3000 years [22]. To adapt to chronic hypoxia, Chinese Tibetans exhibit important physiological and morphologic characteristics of the cardiopulmonary, system such as large chest dimensions [23,24]. A recent report suggested that children and adolescents aged 7–18 years residing above 3500 m in Tibet displayed lower CRF compared with their counterparts from the plains area [25]. Therefore, it is unreasonable to adopt the Chinses national standard to evaluate the CRF of Tibetan children and adolescents at high altitudes, and it is necessary to establish reference values of CRF for this population.

Given the large Tibetan population living in Tibet, China, the present study aimed to develop sex- and age-specific 20mSRT norms for Chinese Tibetan children and adolescents at high altitudes.

## 2. Materials and Methods

### 2.1. Data Source and Participants

From October to December 2019, the research was conducted in Tibet, China. Participants meeting the following requirements were included in the study: (1) had family that lived in Tibet for two generations or more; (2) both the students and their parents are Tibetan (Zang ethnicity); (3) provided written consent from themselves and their parents; (4) had no physical or mental illness.

A stratified random cluster sampling method was used for the selection of participants in two stages. In the first stage, according to geographical distribution and population distribution, Lhasa (3650 m), Nagqu (4500 m), and Amdo (4700 m) were selected, and 21 public schools were selected from the three cities. In the second stage, according to the school size, 2–8 classes were randomly selected from each grade in the 21 public schools. Students in the selected classes meeting the inclusion criteria were recruited as participants. A total of 4982 Tibetan children and adolescents aged 7–18 were included in this research. After excluding 315 (6.32%) data because of missing information, 4667 data were included in the present study.

This study was approved by the Human Subjects Protection Committee of East China Normal University (HR0782020). To protect the privacy of the participants, the names of participants were numbered, and the data was strictly managed.

### 2.2. Measurement

The 20mSRT test was conducted according to the FitnessGram protocol [26]. The measurement details were provided in our previous publications [21,27]. After watching the video containing the test procedure, the participants were told about the test method and details and required to warm up sufficiently before the test. The 20mSRT test was conducted with four physical education teachers present, all of whom were trained and qualified to conduct the test. The number of completed laps was recorded as the results.

### 2.3. Data Analysis

Completed stages/minutes and speed at the last complete stage (km h^−1^) were calculated according to laps.

The lambda, mu, and sigma method (LMS), which is a widely accepted method in reference centile curves, was used to develop 20mSRT norms (laps, completed stages/minutes, the speed at the last completed stage) for Chinese Tibetan children and adolescents [28,29,30]. Cubic splines were obtained using non-linear regression. Smoothing parameters or equivalent degrees of freedom were used to express the extent of smoothing required. The effective degrees of freedom for 20 m SRT in the present study were 1 (L curve), 4 (M curve), and 3 (S curve) for boys; and 1 (L curve), 3 (M curve), and 3 (S curve) for girls. These analyses were performed using the LMS Chart maker Pro version 2.43 (Institute of Child Health, London) [31].

For the speed at the last completed stage, differences in means between (a) age-matched Chinese Tibetan boys and girls (e.g., 10-year-old boys vs. 10-year-old girls); (b) sex-matched Tibetan children of different ages (e.g., girls aged 9 vs. 10 years) were expressed as standardized effect sizes [31]. In age-matched analysis, the mean speed at the last completed stage of Chinese Tibetan girls was taken as a reference, and a positive effect size indicates that the speed at the last complete stage is higher in boys than girls. In sex-matched Tibetan children and adolescents, the mean speed at the last completed stage of 18 years old Chinese Tibetan boys and girls was taken as the reference, respectively, and a positive effect size indicates that, compared to other age groups, the speed at the last complete stage is lower in the 18-year-old age group. Effect sizes of 0.2, 0.5, and 0.8 or −0.2, −0.5, and −0.8 were used as small, moderate, and large thresholds, respectively [32].

All statistics were processed and analyzed using SPSS 25.0 (IBM, Armonk, NY, USA) software and Graph Pad Prism 8, and the significance level was set at *p* = 0.05. The age was calculated based on the last birthday (for example, 7 years old is 7.0–7.9 years old).

## 3. Results

**Figure 1:**
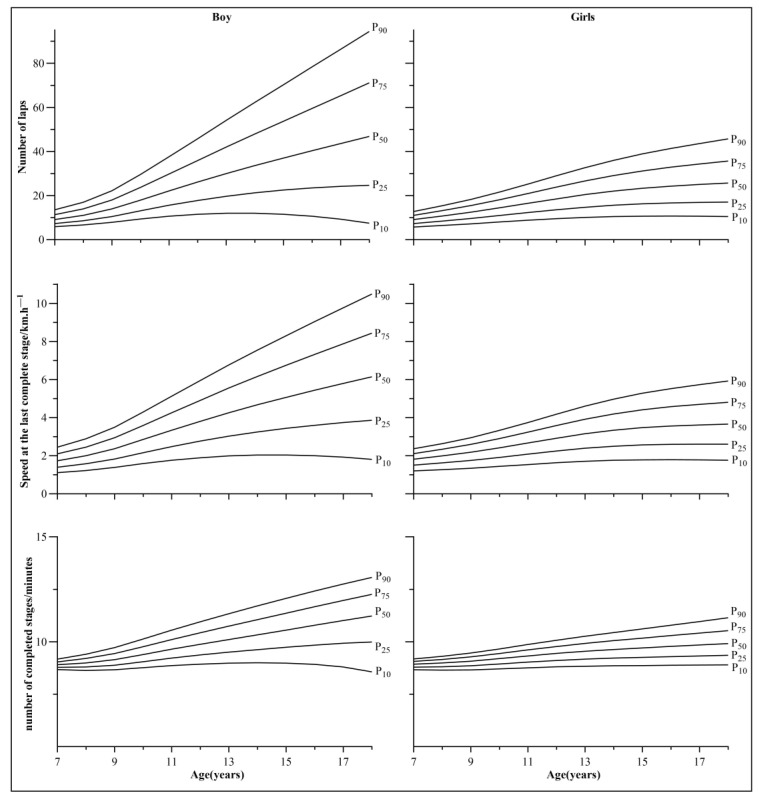
Smoothed centile curves (P10, P25, P50, P75, and P90) of the 20mSRT performance for Chinese Tibetan children and adolescents aged 7–18 in Tibet, China.

Table 1, Table 2 and Table 3 and Figure 1 show the laps, completed stages/minutes, and the speed at the last complete stage percentile values of the 20mSRT for 7–18 years old Chinese Tibetan children and adolescents at high altitudes (P5, P10, P20, P30, P40, P50, P60, P70, P80, P90, and P95). It can be seen that the laps, completed stages/minutes, and the speed at the last complete stage of the 20-mSRT for children and adolescents aged 7–18 increased with age.

**Figure 2:**
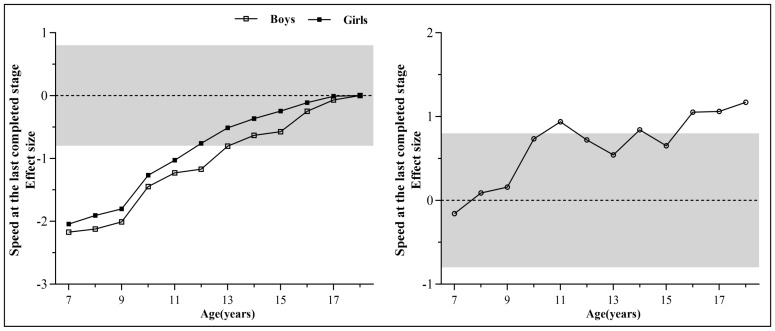
Standardized differences in mean 20mSRT performance (the speed at the last completed stage, km h^−1^). Note: Between sex-matched Chinese Tibetan children and adolescents of different ages (anchored to age 18 years = 0) and age-matched Chinese Tibetan boys and girls. Positive differences indicate that 20mSRT performances were higher for older children than for younger children or for boys than for girls. The limits of the grey zone represent the thresholds for a large standardized difference (0.8 or −0.8).

Figure 2 shows the changes in the standardized differences of the mean 20mSRT performance. In sex-matched analysis, the performances of boys at 7–13 years old and girls at 7–11 years old were significantly lower than that of the 18-year-old age group (the effect size > 0.8). In age-matched analysis, except for the 7-year-old age group, the performances of boys were higher than that of girls, with a significant difference at the ages of 11, 14, 16–18 years (the effect size > 0.8).

## 4. Discussion

Using a representative sample, the present study provided up-to-date age and specific reference data of the number of laps, number of completed stages/minutes, and speed (km h^−1^) at the last complete stage for Chinese Tibetan children and adolescents living at high altitude in Tibet, China. In general, the 20 m SRT performance of Chinese Tibetan children and adolescents aged 9–17 years is lower than the Chinese and international values, especially in the younger age groups.

The CRF of Tibetan children and adolescents aged 9–17 in high-altitude areas of China increases with age, and boys showed higher performance levels than girls, which is consistent with the results of many previous studies. There are many reasons for this result. First, there are congenital genetic differences between Tibetan boys and girls. Compared with girls, boys’ muscle strength and physical activity levels are higher, which is an important reason for boys’ higher CRF than girls [33]. Second, due to the influence of puberty, there are differences in the secretion of hormones between boys and girls. With the advent of puberty, there is also a close relationship between the secretion of male androgen and female estrogen and CRF [34]. Third, performance is also influenced by additional male and female physiological factors. For example, there are certain differences in lean body mass and fat changes, bone growth, etc., between boys and girls, and these factors can also lead to gender differences in CRF [34].

The advantage of this study is that, firstly, this study adopts the international common CRF test method for testing, which is convenient for international horizontal and vertical comparison. Secondly, this study is the first to study the reference value of CRF in high-altitude Tibetan children and adolescents, which provides a reference for the CRF evaluation of Tibetan children and adolescents in high-altitude areas, which is of great interest. However, this study also has some limitations. On the one hand, this study is a cross-sectional study. If we understand the gender and age changes of CRF in Chinese Tibetan children and adolescents, we need to conduct a longitudinal cohort study. On the other hand, although the participants were given positive encouragement in the test performed in this study, there may be deviations between the CRF test value and the real value due to the influence of each participant’s personal subjective will and psychological condition.

## 5. Conclusions

This study developed, for the first time, CRF reference data for Chinese Tibetan children and adolescents in high-altitude areas. Considering the importance of CRF for children and adolescents’ health, the government should strengthen the monitoring of CRF in Tibetan children and adolescents in high-altitude areas, strengthen the reform of the physical education curriculum, and increase the level of physical activity, with the aim is to promote the health level of Chinese Tibetan children and adolescents in high altitude areas.

## Figures and Tables

**Table 1:** Percentile values of laps for Chinese Tibetan children and adolescents aged 7–18 in Tibet, China.

Age (Year)	P5	P10	P20	P30	P40	P50	P60	P70	P80	P90	P95
Boy											
7	5	6	7	8	8	9	10	11	12	14	15
8	6	7	8	9	10	11	12	13	15	17	19
9	6	8	10	11	13	14	15	17	19	22	25
10	7	9	12	14	16	18	20	22	25	30	33
11	8	11	14	17	20	22	25	28	32	38	43
12	8	12	16	20	23	26	30	34	38	46	52
13	8	12	17	22	26	30	34	39	45	54	62
14	7	12	19	24	29	34	39	45	52	62	71
15	6	12	19	26	31	37	43	50	58	70	81
16	5	11	20	27	34	40	47	55	65	78	90
17	2	9	20	28	36	44	52	60	71	86	100
18	0	7	20	29	38	47	56	66	77	94	109
Girls											
7	5	6	7	8	8	9	10	11	12	13	14
8	5	6	8	9	10	11	12	13	14	15	17
9	6	7	9	10	11	12	14	15	16	18	20
10	6	8	10	12	13	14	16	17	19	22	24
11	7	9	11	13	15	16	18	20	22	25	28
12	7	10	12	15	17	19	20	23	25	29	32
13	8	10	13	16	18	20	23	25	28	33	36
14	8	10	14	17	20	22	25	27	31	36	40
15	8	11	15	18	21	23	26	29	33	39	44
16	8	11	15	18	21	24	27	31	35	41	47
17	7	11	15	19	22	25	28	32	37	44	49
18	7	11	15	19	22	26	29	33	38	46	52

**Table 2:** Percentile values of the completed stages/minutes for Chinese Tibetan children and adolescents aged 7–18 in Tibet, China.

Age (Year)	P5	P10	P20	P30	P40	P50	P60	P70	P80	P90	P95
Boy											
7	0.95	1.11	1.31	1.47	1.60	1.73	1.87	2.02	2.20	2.45	2.67
8	1.03	1.23	1.48	1.67	1.84	2.00	2.17	2.35	2.57	2.89	3.16
9	1.14	1.39	1.71	1.95	2.16	2.37	2.58	2.82	3.10	3.50	3.84
10	1.27	1.59	2.00	2.31	2.59	2.85	3.12	3.42	3.78	4.29	4.73
11	1.36	1.76	2.28	2.67	3.01	3.34	3.68	4.05	4.50	5.13	5.67
12	1.41	1.90	2.52	2.99	3.41	3.81	4.22	4.66	5.19	5.95	6.59
13	1.41	1.99	2.74	3.30	3.79	4.26	4.74	5.26	5.89	6.77	7.52
14	1.36	2.04	2.91	3.56	4.13	4.68	5.23	5.83	6.54	7.55	8.40
15	1.25	2.04	3.05	3.80	4.45	5.07	5.69	6.37	7.17	8.30	9.25
16	1.09	2.01	3.16	4.01	4.74	5.44	6.14	6.90	7.79	9.04	10.08
17	0.87	1.93	3.24	4.20	5.02	5.80	6.58	7.41	8.40	9.77	10.90
18	0.58	1.81	3.30	4.37	5.29	6.15	7.01	7.92	9.00	10.49	11.72
Girls											
7	1.01	1.20	1.43	1.58	1.71	1.82	1.93	2.05	2.19	2.37	2.51
8	1.04	1.27	1.53	1.71	1.86	1.99	2.12	2.26	2.42	2.64	2.81
9	1.08	1.35	1.65	1.85	2.03	2.19	2.34	2.51	2.69	2.95	3.16
10	1.14	1.44	1.79	2.03	2.23	2.42	2.60	2.80	3.02	3.33	3.58
11	1.20	1.54	1.94	2.22	2.45	2.67	2.89	3.12	3.38	3.75	4.05
12	1.26	1.63	2.08	2.40	2.67	2.92	3.17	3.44	3.75	4.18	4.54
13	1.30	1.71	2.21	2.56	2.87	3.15	3.44	3.75	4.11	4.60	5.02
14	1.33	1.76	2.30	2.69	3.02	3.34	3.66	4.00	4.41	4.98	5.45
15	1.34	1.79	2.35	2.77	3.13	3.47	3.82	4.20	4.65	5.28	5.81
16	1.34	1.79	2.37	2.81	3.19	3.56	3.94	4.35	4.83	5.53	6.11
17	1.33	1.78	2.37	2.83	3.23	3.62	4.02	4.45	4.98	5.73	6.38
18	1.31	1.77	2.37	2.83	3.25	3.66	4.08	4.55	5.11	5.93	6.63

**Table 3:** Percentile values of the speed at the last complete stage (km h^−1^) for Chinese Tibetan children and adolescents aged 7–18 in Tibet, China.

Age (Year)	P5	P10	P20	P30	P40	P50	P60	P70	P80	P90	P95
Boy											
7	8.61	8.68	8.76	8.81	8.87	8.91	8.96	9.02	9.08	9.18	9.26
8	8.54	8.64	8.76	8.84	8.92	9.00	9.08	9.16	9.26	9.41	9.54
9	8.54	8.67	8.83	8.95	9.05	9.16	9.26	9.38	9.52	9.73	9.91
10	8.61	8.77	8.97	9.13	9.27	9.40	9.53	9.69	9.87	10.14	10.37
11	8.67	8.87	9.13	9.32	9.49	9.65	9.82	10.01	10.23	10.56	10.85
12	8.69	8.94	9.25	9.49	9.69	9.89	10.09	10.31	10.58	10.96	11.30
13	8.68	8.99	9.36	9.64	9.88	10.12	10.35	10.61	10.91	11.35	11.72
14	8.63	9.00	9.46	9.79	10.07	10.34	10.61	10.90	11.24	11.72	12.12
15	8.53	8.99	9.54	9.93	10.26	10.57	10.87	11.19	11.57	12.08	12.50
16	8.35	8.93	9.60	10.06	10.45	10.79	11.13	11.48	11.89	12.43	12.86
17	8.03	8.81	9.64	10.19	10.63	11.02	11.39	11.77	12.20	12.76	13.21
18	7.46	8.57	9.64	10.30	10.80	11.24	11.65	12.06	12.50	13.08	13.53
Girls											
7	8.59	8.67	8.76	8.83	8.88	8.94	8.99	9.04	9.10	9.18	9.25
8	8.55	8.65	8.77	8.86	8.93	8.99	9.06	9.12	9.20	9.31	9.39
9	8.54	8.66	8.81	8.91	9.00	9.08	9.16	9.24	9.34	9.47	9.57
10	8.57	8.71	8.88	9.01	9.11	9.20	9.30	9.40	9.51	9.67	9.79
11	8.60	8.76	8.96	9.10	9.22	9.33	9.44	9.56	9.69	9.88	10.03
12	8.63	8.81	9.03	9.19	9.33	9.45	9.58	9.71	9.86	10.08	10.25
13	8.64	8.84	9.09	9.26	9.41	9.55	9.69	9.85	10.02	10.27	10.47
14	8.64	8.86	9.12	9.32	9.48	9.64	9.80	9.97	10.17	10.45	10.68
15	8.64	8.87	9.16	9.36	9.54	9.72	9.89	10.08	10.30	10.62	10.89
16	8.65	8.88	9.18	9.41	9.60	9.79	9.98	10.18	10.43	10.79	11.10
17	8.65	8.89	9.21	9.44	9.65	9.85	10.06	10.29	10.57	10.97	11.32
18	8.65	8.91	9.23	9.48	9.70	9.92	10.14	10.39	10.70	11.16	11.56

## Data Availability

To protect the privacy of participants, the questionnaire data will not be disclosed to the public. If necessary, you can contact the corresponding author.
